# Non-uniform model of relationship between surface strain and rust expansion force of reinforced concrete

**DOI:** 10.1038/s41598-021-88146-2

**Published:** 2021-04-22

**Authors:** Fan-xiu Chen, Yi-chen Zhong, Xin-ya Gao, Zu-quan Jin, En-dong Wang, Fei-peng Zhu, Xin-xing Shao, Xiao-yuan He

**Affiliations:** 1 School of Science, Qingdao University of Technology, Fushun Road 11#, Qingdao, 266033 China; 2 School of Civil Engineering, Qingdao University of Technology, Qingdao, 266033 China; 3 College of Mechanics and Materials, Hohai University, Nanjing, 211100 China; 4 Department of Engineering Mechanics, Southeast University, Nanjing, 210096 China; 5 Sustainable Construction, State University of New York, Syracuse, NY 13210 USA

**Keywords:** Structural materials, Civil engineering

## Abstract

When operating within the environments rich with sodium chloride, steel bars of reinforced concrete structures are often subject to corrosion caused by surrounding erosive materials, and the associated rust expansion force due to corrosion takes a critical role in determining the durability of relevant reinforced concrete structures. By investigating the corrosion course of steel reinforcement with theory of elasticity, a numerical rust expansion model is established for the moment of concrete surface rupture based on non-uniform sin function. Cuboid reinforced concrete specimen with squared cross sections is tested to analyze the rust expansion when concrete cracks due to corrosive forces. The utility of the established expansion model is validated by numerical simulation with Abaqus through the comparison between the associated outcomes. The impacts of steel bar diameter and concrete cover thickness on the magnitude of rust expansion force are discussed.

## Introduction

The presence of chlorides in marine environments, e.g. coastal regions, remains one of the leading drivers of corrosion of steel bars embedded in reinforced concrete structures^1^. While corrosion courses and behaviors attributed to chloride corrosives can dramatically vary due to diverse service environments and different rebar sections, in general, when rebar corrosion occurs, the bond strength between concrete and steel bars often decreases significantly when a certain level of rust accumulation reaches. Meanwhile, volume expansion as a consequence of rust accumulation, can result in serious rupture failure of concrete elements^2^. After concrete cracks, rebar corrosion will be exacerbated due to the increased exposure of more reinforcements to surrounding humid conditions. Eventually, the durability of the associate components and even the entire structures can be severely damaged^3,4^.

In order to understand the nonlinear behavior of reinforced concrete beam under corrosion during fatigue loading process, a piecewise linear model was proposed by Zhang et al.^5^ for simulation purpose. Experiments results were used for model verification. Mir et al.^6^ proposed an enhanced numerical forecast model for predicting service life of reinforced concrete structure by considering chloride and oxygen concentrations. Amey et al.^7^ introduced an environmental approach for estimating the possible service life of concrete structures in different operation circumstances. Several potential influencers covering surface conditions, chloride movements, medium temperature, seasonal impacts, and construction differences, were considered during the prediction process. Multiple concrete mixtures at diverse quality levels were assed in their illustrations.

Using digital microscopy technique, Ye et al.^8^ explored the patterning of bar corrosion and the cracking mechanism of concrete cover for corner reinforcement. Gaussian models were adopted for characterizing dynamic propagation patterns of rust relative to corroded bar positions. German and Pamin^9^ employed the damage-plasticity model of Abaqus on the basis of finite element method to study reinforcement corrosion. In case of multiple steel bars, if concrete cover is limited, fractures often occur between reinforcements and concrete cover. Consequently, moisture and chloride can easily reach reinforcements and exacerbate corrosion. When concrete cover is relatively thick, fractures often only appear between reinforcements.

Yuan and Ji^10^ showed that reinforcement corrosion often presents more seriously on one specific face and then gradually reduces on other faces. To research the crack development due to corrosion in concrete, Chen^11^ invented finite element model and boundary element model. Non-uniform distribution of rust around bar cross sections was taken into account which presented a more realistic reflection on the practical situation of chlorides penetrating through member covers to surfaces. Yang et al.^12^ demonstrated a dynamic corrosion process model considering non-uniform expansion of reinforcement. Complex functions were applied to calculate stresses in concrete. The starting time of concrete fracturing was decided by relating material, geometry and corrosion properties.

Concrete cracking due to reinforcement corrosion stands one of the major drivers of concrete structure failure. Reinforcement corrosion course is often non-uniform along bar circumference. To characterize the stress distribution in concrete and determine initiation of concrete cracking, more realistic models for describing non-uniform corrosion courses are substantially demanded. This paper proposes a reinforcement corrosion model with sine theory to simulate the corrosion scenario where all the rebar sections are under corrosion and the face exposed to concrete cover has more serious corrosion. The rebar rust expansion force at the moment of cracking is derived. It analyzes such common parameters as rebar diameter, expansivity of corrosion products and thickness of concrete cover, and the impacts of individual parameters on rust expansion force by comparison.

## Non-uniform reinforcement corrosion model

**Figure 1:**
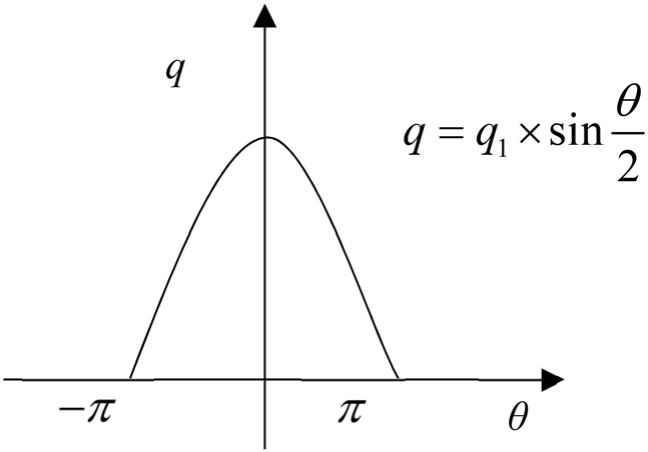
Sine function curve of rust force.

**Figure 2:**
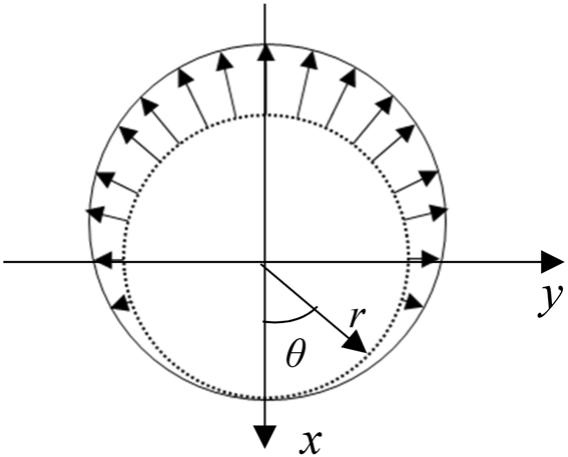
Rust force pattern.

For model construction, it assumes that when steel bars corrode, with the Cartesian coordinate system, the thickness of rust products displays in sine function pattern. Rust products are presumed to expand over the steel bars and exert forces outwards to surrounding concrete components. Rust products are subject to constraint pressure from concrete and steel bars and the distribution pattern of the corresponding rust force follows sine function curve, as shown in Figs. 1 and 2 where *q* means rust force and *q*_1_ represents error reinforcement rust force.

**Figure 3:**
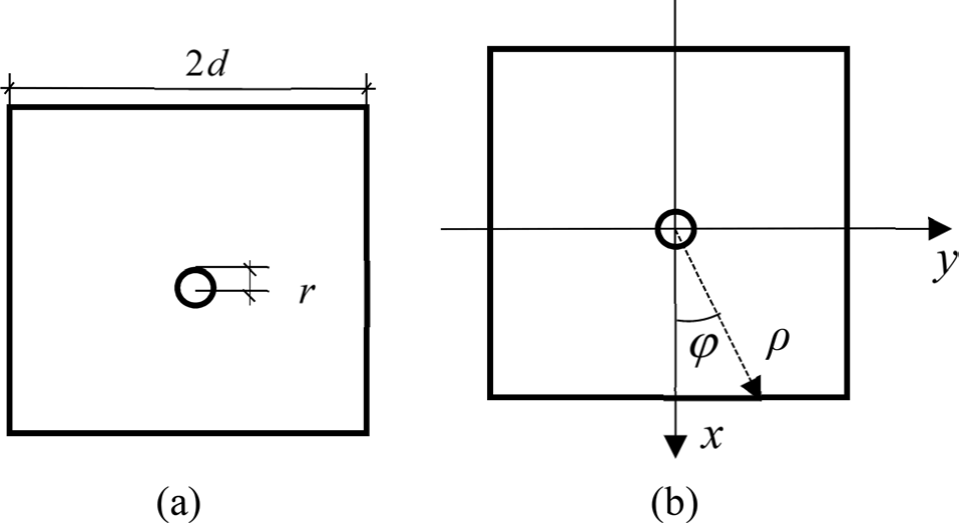
Specimen sketch. (**a**) Schematic diagram of specimen; (**b**) establishment of coordinate system.

When creating non-uniform sine function rust expansion force model, to simplify the computation course, fundamental elasticity theory^12^ is followed for the analysis with the sketch of computation shown in Fig. 3. The cross section of concrete specimen is a square with side length 2*d*, and the radius of reinforcement is *r*. Taking the concrete part as the research object, the coordinate system is established with the center of the steel bar as the origin of the coordinate axis, as shown in Fig. 3b.

Reinforced concrete specimen is divided into reinforcement and concrete sections for calculation by the theory of mechanics of elastic contacts. In Cartesian coordinate system, the outline of rust expansion force due to reinforcement corrosion follows sine function which can be used to characterize the approximation of the corresponding distribution pattern as in Eq. (1).

1 $$ q = q_{1} \times \sin \frac{\theta }{2} $$

where *q* means rust force and *q*_*1*_ represents error reinforcement rust force.

Concrete section is taken as research object and with the center of circular hole as its origin, the polar coordinate system is set up. Stress function Φ is assumed as Eq. (2) and A. B and C are the coefficients to be solved.

2 $$ \Phi \left( {\rho ,\varphi } \right) = \left( {A\rho + B + C\frac{1}{{\rho^{2} }}} \right)q\sin \frac{\varphi }{2} $$

Stress components are obtained through Eq. (2):

$$ \begin{aligned} \sigma_{\rho } & = \frac{1}{\rho }\frac{\partial \Phi }{{\partial \rho }} + \frac{1}{\rho }\frac{{\partial^{2} \Phi }}{{\partial \varphi^{2} }} = \left( {\frac{3}{4}\frac{A}{\rho } - \frac{1}{4}\frac{B}{{\rho^{2} }} - \frac{9}{4}\frac{C}{{\rho^{4} }}} \right)q\sin \frac{\varphi }{2} \\ \sigma_{\varphi } & = \frac{{\partial^{2} \Phi }}{{\partial \rho^{2} }} = 6\frac{C}{{\rho^{4} }}q\sin \frac{\varphi }{2} \\ \tau_{\rho \varphi } & = - \frac{\partial }{\partial \rho }\left( {\frac{1}{\rho }\frac{\partial \Phi }{{\partial \varphi }}} \right) = - \frac{1}{2}\left( {\frac{B}{{\rho^{2} }} + 4\frac{C}{{\rho^{4} }}} \right)q\cos \frac{\varphi }{2} \\ \end{aligned} $$

where *Ϭρ* and *Ϭφ* means radial stress and circumferential stess, *τ*_*ρφ*_ is tangential stress.

Boundary conditions are shown as the following:

$$ \begin{aligned} & (\sigma_{\rho } )_{\rho = r} = - q_{1} \sin \frac{\theta }{2} \\ & (\sigma_{\rho } )_{\rho = d} = 0 \\ & (\tau_{\rho \varphi } )_{\rho = r} = 0 \\ & (\sigma_{\varphi } )_{\rho = d,\varphi = 0} = 0 \\ \end{aligned} $$

Stress components are eventually derived:

3 $$ \begin{aligned} \sigma_{\rho } & = \left( {\frac{9}{{4\rho d^{3} }} - \frac{3}{{\rho dr^{2} }} + \frac{1}{{\rho r^{2} }} - \frac{9}{{4\rho^{2} }}} \right)\left( {\frac{{4q_{1} r^{4} d^{3} }}{{4rd^{2} + 5d^{3} - 9r^{3} }}} \right)\sin \frac{\varphi }{2} \\ \sigma_{\varphi } & = \frac{6}{{\rho^{4} }}\frac{{4q_{1} r^{4} d^{3} }}{{4rd^{2} + 5d^{3} - 9r^{3} }}\sin \frac{\varphi }{2} \\ \tau_{\rho \varphi } & = \left( {\frac{2}{{\rho^{2} r^{2} }} - \frac{2}{{\rho^{4} }}} \right)\left( {\frac{{4q_{1} r^{4} d^{3} }}{{4rd^{2} + 5d^{3} - 9r^{3} }}} \right)\cos \frac{\varphi }{2} \\ \end{aligned} $$

## Model validation

### Theoretical validation

Due to rust expansion forces, the interface concrete undergoes outward radial displacement on all the cross sections. The thickness of rust products on the interface between concrete and reinforcement equals the summation of the radial displacement of concrete and the depth of reinforcement corrosion. That is, on the interface between concrete and reinforcement, the deformation compatibility should be satisfied with radial displacements.

4 $$ \varepsilon_{\rho } = \frac{1}{E}\left( {\sigma_{\rho } - \mu \sigma_{\varphi } } \right) $$

5 $$ \varepsilon_{\rho } = \frac{{\partial u_{\rho } }}{\partial \rho } $$

where E is the modulus of elasticity and μ is the Poisson's ratio, *u*_*ρ*_ is displacement at the point *ρ* in the direction of radius.

Radial displacement of concrete can be obtained:

6 $$ u_{\rho } = \frac{1}{{E_{c} }}\left[ {\left( {\frac{9}{{4d^{3} }} - \frac{3}{{dr^{2} }} + \frac{1}{{r^{2} }}} \right)\ln \rho + \frac{9}{4\rho } + \mu \frac{2}{{\rho^{3} }})} \right]\left( {\frac{{4q_{1} r^{4} d^{3} }}{{4rd^{2} + 5d^{3} - 9r^{3} }}} \right)\sin \frac{\varphi }{2} $$

Compatibility of deformations on the interfaces (at r = *ρ*) between bars and concrete is considered. In one unit length of reinforced concrete, the displacement due to rust products is equal to the radial displacement of concrete due to rust expansion force *q*. Then the below can be established.

7 $$ \left( {n - 1} \right)\eta \pi r^{2} = \int_{0}^{2\pi } {\frac{{(r + (u_{\rho } )_{\rho = r} )^{2} - r^{2} }}{2}d\varphi } = \int_{0}^{2\pi } {r(u_{\rho } )_{\rho = r} d\varphi } $$

where *n* is volume expansion rate of corrosion products and is generally 2–4; *η* is corrosion rate of reinforcement.

Combining Eqs. (3)–(7), rust expansion force can be computed as:

8 $$ q_{1} { = }\frac{{E_{c} \left( {n - 1} \right)\eta \pi r^{2} }}{{\left[ {\left( {\frac{9}{{4d^{3} }} - \frac{3}{{dr^{2} }} + \frac{1}{{r^{2} }}} \right)\left( {\frac{{r^{2} }}{2} - \frac{{r^{2} }}{2}\ln r} \right) - \frac{9}{4}\ln r - \mu \frac{1}{{r^{2} }}} \right]\left( {\frac{{4r^{4} d^{3} }}{{4rd^{2} + 5d^{3} - 9r^{3} }}} \right)}} $$

Based on the Code for Design of Concrete Structures (GB50010-2010), the elasticity modulus of concrete Ec and Poisson’s ratio are respectively set to 2.8 × 10^4^ N/mm^2^ and 0.2. For theoretical validation, using the values r = 8 mm, d = 23 mm from previous literature^13^, by the above Eq. (8), when the concrete protective layer is 15 mm, the obtained rust expansion force q corresponding to the moment when concrete cracks, is 1.421 N/mm^2^. This value is comparable to the experimental result which is 1.2 N/mm^2^ in existing literature^14^. It indicates that the model can be effectively applied to the prediction of the magnitude of rust expansion force when concrete cover fails. However, due to the ignorance of plastic deformation during model construction which relies on elasticity theory, the rust expansion force from the established numerical model could be greater than the actual expansion value.

### Numerical simulation using finite element method

**Figure 4:**
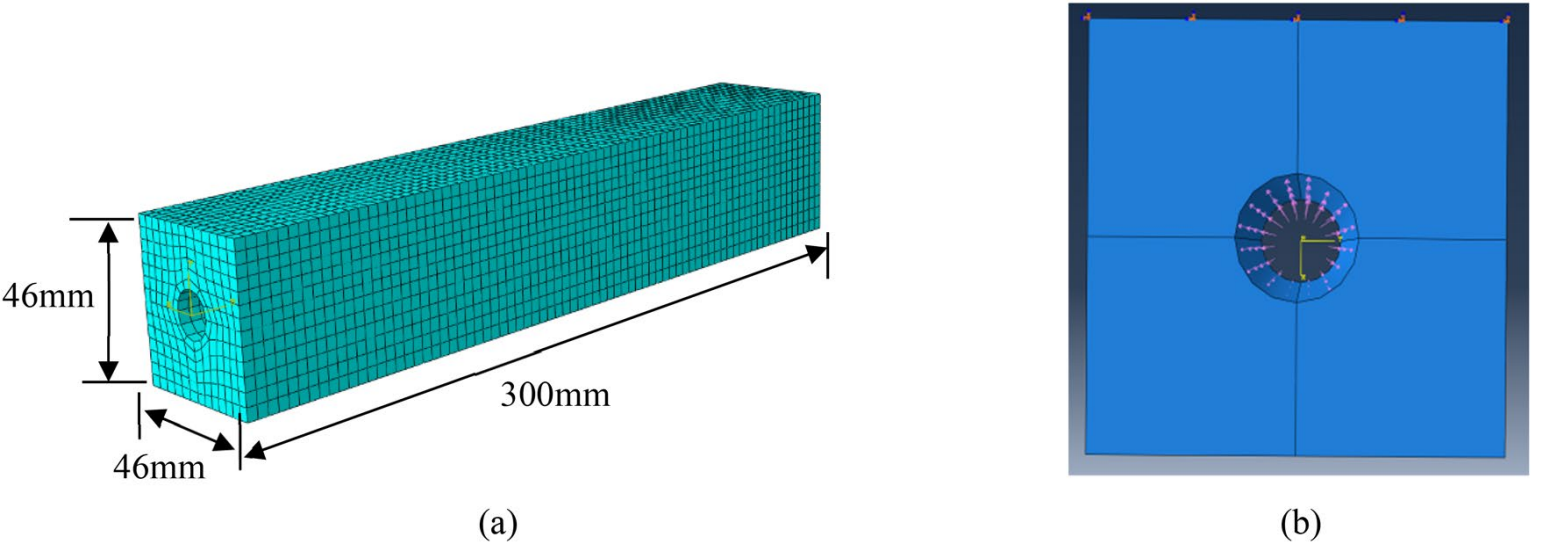
Model setting up. (**a**) Simulation object with meshing. (**b**) Rust expansion force distribution.

Abaqus6.14 software is used for the numerical simulation based on finite element method. A simulation object is set up in the dimensions of 46 mm × 46 mm × 300 mm, with a circular hollow having the section radius of 8 mm. Classical fracture and damage model^15^ is adopted with attribute and parameter values shown in Table 1. The object model with meshing is displayed in Fig. 4a. The expansion forces with distribution pattern shown in Fig. 4b are exerted to the circular hollow walls with the largest value of 5 N/mm^2^ to simulate reinforcement expansion process. Normal displacements associated with the side faces of the established model are relatively small. For modeling convenience, the boundary conditions are defined to constraint the normal displacements of four side faces^16^.

**Table 1:** Concrete property parameters in simulation.

Expansion angle	Eccentricity	fb_0_/fc_0_	K	Elasticity	Poisson’s ratio	Viscosity
30°	0.1	1.16	0.667	2.8 × 10^4^ N/mm^2^	0.2	0.005

f_b0_/f_c0_ is the ratio of biaxial and uniaxial compressive strengths; parameter K determines the yield surface shape and is defined as 2/3.

And ultimate tensile strength is 1.78 N/mm^2^.

**Figure 5:**
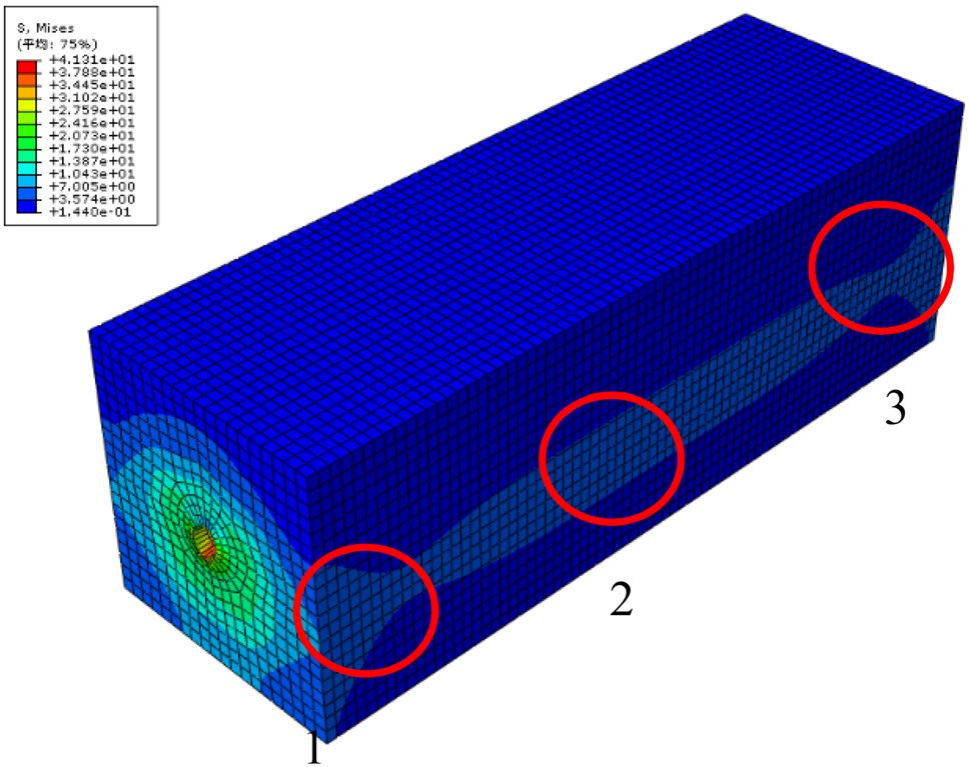
Mess stress in numerical simulation under nonuniform corrosion.

The Mess stress obtained by numerical simulation under non-uniform corrosion is found in Fig. 5. It can be seen that the stress distributions in tip Region 1 and Region 3 differ from that in Region 2 which presents more stable stress profile. Hence, the distribution results on the cross sections in Region 2 are taken for the later analysis.

**Figure 6:**
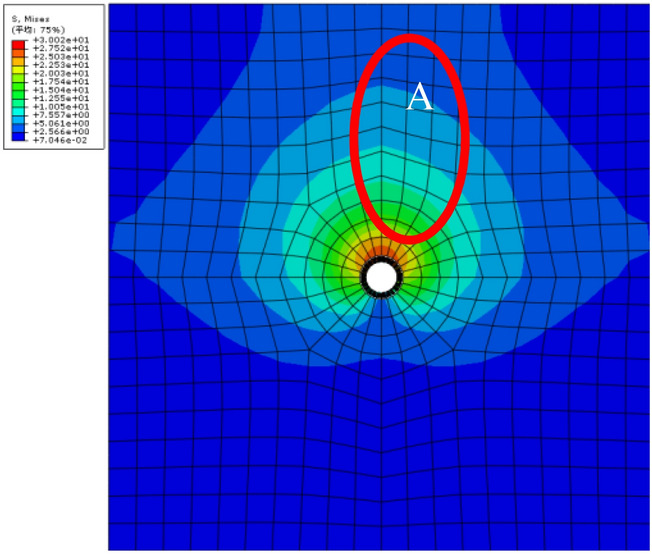
Tensile stress from numerical simulation under non-uniform corrosion.

Figure 6 shows the simulation results, as rust expansion force is increased to 1.74 N/mm^2^. A V-shape expansion pattern is discerned for stress distribution. In Region A, more than half of the tensile stresses are higher than the ultimate tensile strength. Therefore, it can be judged that concrete cracks first appear in Region A, before the expansion force reaches 1.74 N/mm^2^. Since the boundary conditions are set to have zero normal displacements on the four side faces, more expansion forces are required to enable the stress to reach ultimate tensile strength. For this perspective, it should be reasonable to receive a value greater than the experimental result which is 1.2 N/mm^2^ in existing literature^14^.

**Figure 7:**
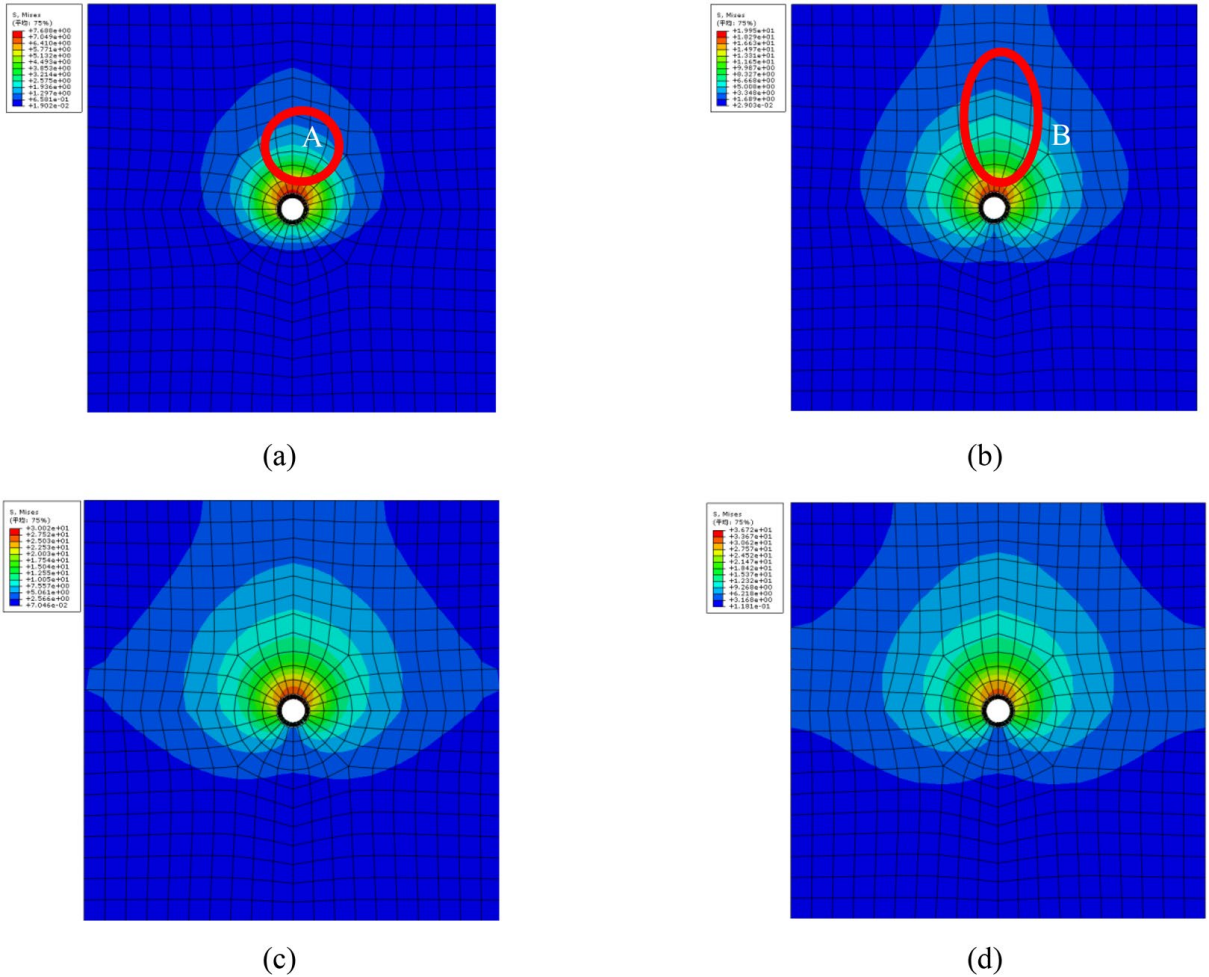
Staged profile of tensile stress in numerical simulation under non-uniform corrosion, (**a**) a stage, (**b**) b stage, (**c**) c stage, (**d**) d stage.

The stress around circular hollow increases with the rise in expansion force, demonstrating a clear layering style. Meanwhile, the outline initiates as being drop-shaped. As the expansion forces increase, the upper tensile stress grows rapidly, while the lower tensile stress develops slowly or even suspends, as shown in Fig. 7a. Responding to the growth in expansion force, the upper area in tension state expands over the model surface. Tensile stress appears on both the left and the right sides of the object model. The tensile stress appears relatively larger around the center region and reduces exponentially along the pathways away from the center region to the surface areas. The tensile stress decreases gradually in the surface regions of the modeled object, as shown in Fig. 7b.

As the rust expansion force grows, the tensile stress first presents in the inner part and gradually expands to the surface regions with a tendency of continual expansion, as in Fig. 7c. However, the stress on the end surface appears stably lower, as in Fig. 7d. From the staged profiles, the stress in B region remains maximum without significant fluctuation. The stress turns higher and eventually leads to cracks in that region. The stress in the area above the hole stays much higher than in the bottom area as in Region A. Overall, the majority of the stress appears on the upper part of the model.

## Discussion on influencers of rust expansion force

### Rebar size

The corrosion of steel bars implanted in concrete negatively impacts the relevant structures by reducing the corresponding durability. Nevertheless, rebar components can positively enhance the corresponding structures in the capacity of resisting bending and tensile loads. Overall, net benefits are generated as the consequence of embedding steel bars in concrete for reinforcement. Currently, in China, multiple types of rebars exist with different diameter sizes of 8 mm,16 mm, 32 mm and 40 mm. Steel bars in distinct diameter sizes possibly exhibit varying corrosion rates along with unsimilar magnitudes of rust expansion force, when concrete cracks. By controlling all the other parameters constant and altering diameter size of steel bars, the corresponding rust expansion forces at the moment of concrete cracking are calculated. The selected bar diameters are 8 mm,16 mm, 20 mm with the respective corrosion rates corresponding to concrete failure being listed in Table 2^14^. It used deformed steel bar in HRB335 with carbon ≤ 0.25%. Theoretical yield strength and tensile strength are greater than 335 N/cm^2^ and 490 N/cm^2^, respectively.

**Table 2:** Bar sizes and corrosion rates.

Rebar diameter (mm)	8	16	20
Corrosion rate at cracking (%)	9.04	4.07	2.3

**Figure 8:**
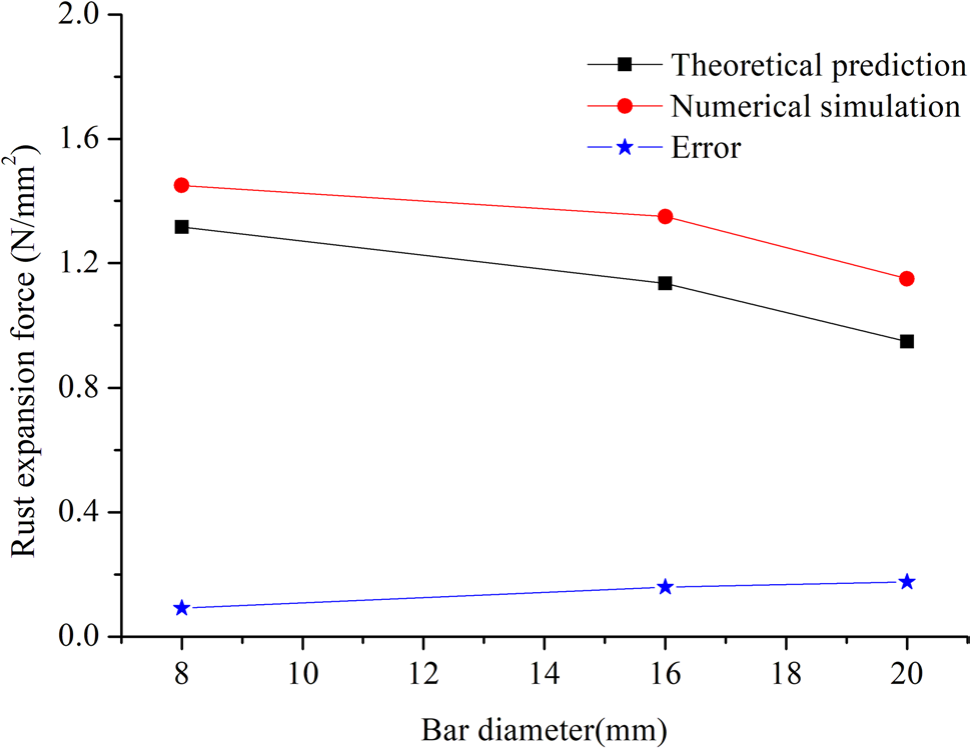
Relationship between rust expansion force and bar diameter.

When concrete cracks, the relationship profiles between rust expansion force and bar diameter size are plotted in Fig. 8, based on the outcomes of theoretical prediction and numerical simulation. Although the differences between theoretical results and simulation results 9.2%, 15.6% and 17.1% for 8 mm, 16 mm and 20 mm bars, respectively, these results have the same rules. The results from both the theoretical prediction and the numerical finite element simulation indicate that, when the other parameters remain the same, the rust expansion force at cracking decreases as bar size increases. When the other parameters remain unchanged, as rebar diameter becomes larger, higher levels of corrosion occur with more bars corroded and then more rust products are generated. Consequently, the growth rate of rust expansion force accelerates and eventually leads to the earlier cracking of concrete protective layer.

### Concrete cover depth

When corrosion occurs to steel bars, for specific reinforced concrete structures, the thicker the concrete cover is, the more rust expansion force it requires to crack the concrete cover. Apparently, from this perspective, the durability and safety of reinforced concrete structures can be enhanced by thickening the associate concrete layer for protection. On the other hand, the protection covers in super depth can lead to over extensive fracture gaps when the relevant structure cracks due to rust expansion. Over extensive cracks on concrete protection layer could cause unnecessary panics among the connected users of concrete structures.

**Figure 9:**
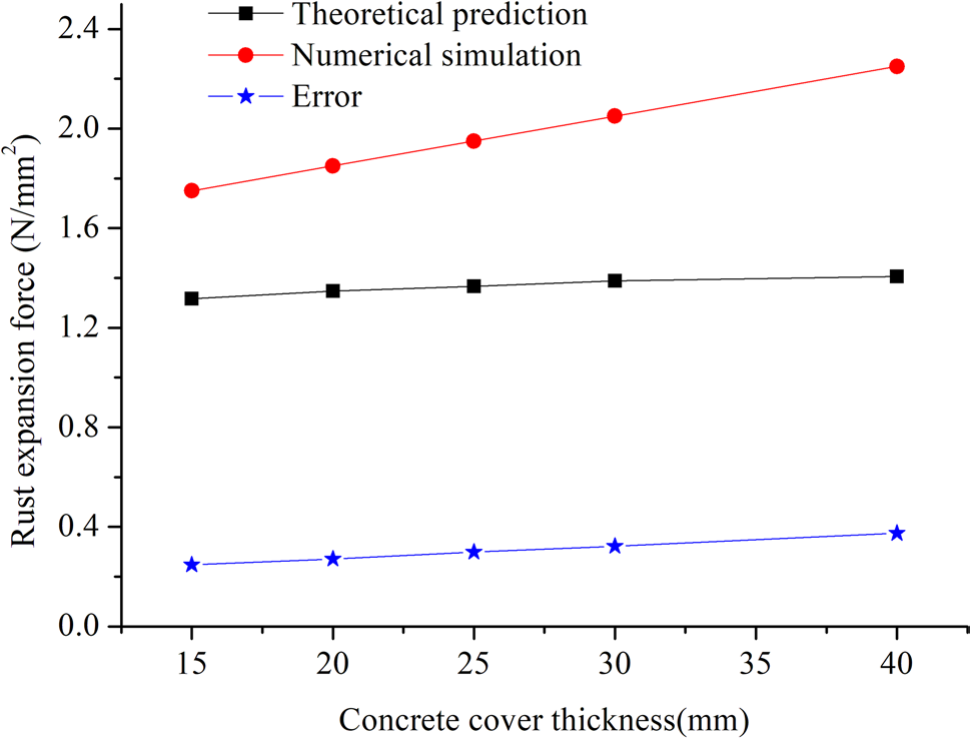
Relationship between rust expansion force and concrete cover thickness.

The relationship between rust expansion force and concrete protection layer under diverse rust expansion rates is presented in Fig. 9 in which both theoretical computation results and numerical simulation outcomes are accounted. In Fig. 9, the minimum difference between the theoretical results and simulation results is 24.7% and the maximum difference is 37.4%. Although there are some errors, the change laws are consistent. The impacts of the changes in three parameters on rust expansion forces are analyzed: bar diameter, corrosion rate and thickness of protection layer. When the impact of one parameter is analyzed, the other two parameters should be fixed. For the relationship investigation, the bar diameter is set to 8 mm and the rust expansion rate is fixed at 2.5. It can be seen, the thickened concrete cover could not necessarily prevent rebar corrosion in concrete to reduce rust expansion forces. Conversely, when the thickness of concrete cover increases, the constraining forces on steel bars originating from concrete cover turn larger. As a result, when rust products appear, the bonding forces imposed to them will be greater and larger rust expansion forces will occur. From this point, to arbitrarily increase the depth of protective layer may not necessarily produce favorable effects to improve structure durability and safety. Therefore, it may be effective to improve the durability and safety of structures and then reduce corrosion-induced cracking risks by thickening concrete cover, but the adoption of this approach should be limited to a certain suitable range. For the same rebar size, different rust expansion force values can be found from theoretical prediction than from numerical simulation. The cause of difference is that, the theoretical approach assumes pure elasticity while ignoring plasticity effects, but numerical simulation incorporates the effects of plasticity.

### Rust product expansion rate

**Figure 10:**
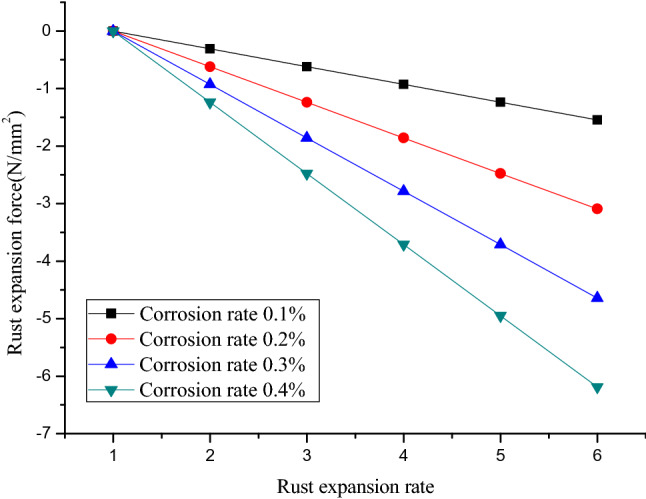
Relationship between rust expansion force and corrosion rate.

For theoretical computation, rust expansion is modelled based on the assumption of perfect elasticity. In addition, the compression course of rust products is neglected in order to simplify the computation process. Then, the expansion rate is actually calculated relative to the volume after compression. From the above formula (8), rust product expansion rate is directly proportional to rust expansion force. That is, the greater the rust product expansion rate becomes, the larger the rust expansion force turns, and vice versa. When bar diameter is 8 mm and concrete cover thickness is 15 mm, the relationship between rust expansion force and expansion rate is plotted against varying corrosion rates in Fig. 10. The corrosion rate is the percentage of the corroded bar mass in related to the raw bar mass.

It can be visually observed from Fig. 10 that, under the same corrosion rate, as rust expansion rate rises, the rebar rust expansion force increases linearly rather than exponentially. It can be deduced that the rust volume expansion rate has lower impacts on rust expansion force than corrosion rate, which is consistent with the existing results in previous literature^17^. According to the impacts of rust volume expansion rate on rust expansion force, it appears feasible to lower down the corrosion-induced rust expansion force in reinforced concrete structures by either adjusting constituents in steel bars or modifying associate processing technologies to reduce rust expansion rate. Unfortunately, few studies related to rust products have been conducted, and the majority of the relevant researchers intensively used the parameter values regarding elasticity modulus, Poisson’s ratio and volume expansion rate of steel rust products from literature^15,16^. If more realistic information on elasticity modulus and Poisson’s ratio was added during the theoretical modeling process, more accurate results on rust expansion force would have been obtained.

## Conclusion

This paper establishes a sine-function-based model to investigate rust expansion process of reinforced concrete cuboid specimen with squared cross sections, along with its verification through numerical simulation by Abaqus using finite element analysis. The conclusions are as follows:

1. From the cross-verification process between theoretical modeling and numerical simulation, comparable results are obtained from the established non-uniform rust expansion model against numerical simulation. While numerical simulation considers plastic deformation phase, it defines boundary conditions to prescribe the lower part of model object to be tensile in normal direction, which makes the simulated rust expansion force larger than that received from analytical modeling process.
2. Through the analyses of the impacts of rebar diameters and concrete cover depth on rust expansion force, when concrete cracks, under the same rebar size and concrete cover depth, numerical simulation produces greater rust expansion force than theoretical modeling process.
